# A pre-existing population of ZEB2^+^ quiescent cells with stemness and mesenchymal features dictate chemoresistance in colorectal cancer

**DOI:** 10.1186/s13046-019-1505-4

**Published:** 2020-01-08

**Authors:** Federica Francescangeli, Paola Contavalli, Maria Laura De Angelis, Silvia Careccia, Michele Signore, Tobias Longin Haas, Federico Salaris, Marta Baiocchi, Alessandra Boe, Alessandro Giuliani, Olga Tcheremenskaia, Alfredo Pagliuca, Ombretta Guardiola, Gabriella Minchiotti, Lidia Colace, Antonio Ciardi, Vito D’Andrea, Filippo La Torre, JanPaul Medema, Ruggero De Maria, Ann Zeuner

**Affiliations:** 1 Department of Oncology and Molecular Medicine, Istituto Superiore di Sanità, Viale Regina Elena 299, 00161 Rome, Italy; 2 Institute of General Pathology, Università Cattolica del Sacro Cuore, Largo Francesco Vito 1, 00168 Rome, Italy; 3 RPPA Unit, Proteomics Area, Core Facilities, Istituto Superiore di Sanità, Viale Regina Elena 299, 00161 Rome, Italy; 4 Fondazione Policlinico Universitario A. Gemelli IRCCS, Largo Agostino Gemelli 8, 00168 Rome, Italy; 5 Core Facilities, Istituto Superiore di Sanità, Viale Regina Elena 299, 00161 Rome, Italy; 6 Environment and Health Department, Istituto Superiore di Sanita, Viale Regina Elena 299, 00161 Rome, Italy; 7 Stem Cell Fate Laboratory, Institute of Genetics and Biophysics “A. Buzzati Traverso”, CNR,Via Pietro Castellino 111, 80131 Naples, Italy; 8 Department of Surgical Sciences, Policlinico Umberto I/Sapienza University of Rome, Viale del Policlinico 155, 00161 Rome, Italy; 9 Department of Surgery “Pietro Valdoni”, Policlinico Umberto I/Sapienza University of Rome, via Lancisi 2, 00161 Rome, Italy; 10 Surgical Sciences and Emergency Department, Policlinico Umberto I/Sapienza University of Rome, Viale del Policlinico 155, 00161 Rome, Italy; 11 Laboratory for Experimental Oncology and Radiobiology, Center for Experimental and Molecular Medicine, Cancer Center Amsterdam, Academic Medical Center, Meibergdreef 9, 1105 AZ Amsterdam, The Netherlands

**Keywords:** Colorectal cancer, Chemotherapy resistance, Dormancy, Quiescence, Epithelial-to-mesenchymal transition, Cancer stem cells

## Abstract

**Background:**

Quiescent/slow cycling cells have been identified in several tumors and correlated with therapy resistance. However, the features of chemoresistant populations and the molecular factors linking quiescence to chemoresistance are largely unknown.

**Methods:**

A population of chemoresistant quiescent/slow cycling cells was isolated through PKH26 staining (which allows to separate cells on the basis of their proliferation rate) from colorectal cancer (CRC) xenografts and subjected to global gene expression and pathway activation analyses. Factors expressed by the quiescent/slow cycling population were analyzed through lentiviral overexpression approaches for their ability to induce a dormant chemoresistant state both in vitro and in mouse xenografts. The correlation between quiescence-associated factors, CRC consensus molecular subtype and cancer prognosis was analyzed in large patient datasets.

**Results:**

Untreated colorectal tumors contain a population of quiescent/slow cycling cells with stem cell features (quiescent cancer stem cells, QCSCs) characterized by a predetermined mesenchymal-like chemoresistant phenotype. QCSCs expressed increased levels of ZEB2, a transcription factor involved in stem cell plasticity and epithelial-mesenchymal transition (EMT), and of antiapototic factors pCRAF and pASK1. ZEB2 overexpression upregulated pCRAF/pASK1 levels resulting in increased chemoresistance, enrichment of cells with stemness/EMT traits and proliferative slowdown of tumor xenografts. In parallel, chemotherapy treatment of tumor xenografts induced the prevalence of QCSCs with a stemness/EMT phenotype and activation of the ZEB2/pCRAF/pASK1 axis, resulting in a chemotherapy-unresponsive state. In CRC patients, increased ZEB2 levels correlated with worse relapse-free survival and were strongly associated to the consensus molecular subtype 4 (CMS4) characterized by dismal prognosis, decreased proliferative rates and upregulation of EMT genes.

**Conclusions:**

These results show that chemotherapy-naive tumors contain a cell population characterized by a coordinated program of chemoresistance, quiescence, stemness and EMT. Such population becomes prevalent upon drug treatment and is responsible for chemotherapy resistance, thus representing a key target for more effective therapeutic approaches.

## Background

The existence of cancer cells able to survive antineoplastic drugs and to regenerate a local or distant tumor undermines the effectiveness of cancer therapies. Drug resistance is tightly connected to the presence of cancer stem cells (CSCs) responsible for tumor progression, metastatization and recurrence [1, 2]. Therapy-resistant cells with features of stalled/delayed cycling have been identified in solid and hematologic tumors including melanoma, glioblastoma, medulloblastoma, leukemia, lung, breast, pancreatic and ovarian cancer [3–14], suggesting that a population of quiescent/slow proliferating cancer stem cells (QCSCs) may represent an essential tool by which tumors resist to external challenges. Additionally, quiescence is typical also of tumor cells present in the bloodstream, disseminated in the bone marrow or within lymph nodes (that altogether account for minimal residual disease), suggesting that quiescent cells represent a crucial therapeutic target [15]. In colorectal cancer (CRC), QCSCs were identified as cells able to reactivate upon serial transplantation [16, 17], to survive chemotherapy and to endure metabolic stress [18, 19]. Recently, two distinct populations of slow cycling cells were identified in CRC with different strategies. A label-retaining approach identified dormant CRC cells as a differentiated population with enhanced clonogenic capacity and high levels of Wnt and Hedgehog signaling [20]. Differently, a histone 2B-GFP (H2B-GFP) pulse-chase approach identified a population of slow cycling cells characterized by expression of the TET2 dioxygenase and by enhanced chemoresistance [21]. The quiescent/drug resistant state in solid tumors is tightly linked to tumor heterogeneity and in particular to the ability of cancer cells to undergo epithelial-to-mesenchymal transition (EMT), an epigenetic programme that crucially regulates the stemness, chemoresistance and invasive ability of cancer cells [22]. According to its pleiotropic effects on cellular phenotype and function, EMT recruits a series of genes with multiple functions in embryogenesis and carcinogenesis such as ZEB1, ZEB2, SNAI1, SNAI2 and TWIST1 [23]. Among these, ZEB2 has been shown to regulate epithelial cell plasticity and proliferation, but also to balance stemness and differentiation, standing as a master regulator of cell state transitions [24–26]. Notably, ZEB2 was also recently recognized as a factor implicated in drug resistance in CRC through FBXW7 E3-ubiquitin ligase binding [27]. Moreover, ZEB2 expression was associated with poor oncologic outcome and distant recurrence, emerging as a new clinical biomarker in CRC [28]. In this study, we aimed to isolate and characterize a population of cells with combined features of quiescence and therapy resistance that is present in untreated colorectal tumors and becomes largely prevalent upon chemotherapy treatment. In line with our previous studies showing that PKH-retaining tumor cells were endowed with higher tumorigenic capacity and chemotherapy resistance [14, 16], we undertook an in-depth molecular characterization of PKH26^+^ cells isolated from CRC xenografts through gene expression analysis and reverse-phase proteomic arrays, providing for the first time a combined picture of both transcriptional circuits and activated protein pathways. New insights on the molecular factors that orchestrate quiescence programs will likely open new therapeutic avenues to eradicate non-proliferating cancer cells, both in primary tumors and at premetastatic sites.

## Materials and methods

### Primary colorectal cancer cells and cell lines

Colorectal cancer (CRC) specimens were obtained from patients undergoing surgical resection upon informed consent and approval by the Sapienza-Policlinico Umberto I Ethical Committee (RIF.CE: 4107 17/10/2016). Tissue samples were collected by a pathologist immediately after surgery, quickly washed 2–3 times in cold phosphate buffered saline (PBS) and then transferred in Dulbecco’s modified Eagle’s medium (DMEM; Thermo Fisher Scientific) containing 3% penicillin-streptomycin-amphotericin B solution (Lonza) until processing. For tissue dissociation, CRC samples were first washed 3–4 times in PBS, then cut by forceps and/or scalpel in pieces of approximately 0.5 mm or smaller. Fragments were further washed twice by centrifugation at 150 g for 3 min, then incubated in DMEM with 1.5 mg/ml collagenase type II (Thermo Fisher Scientific) and 20 mg/ml DNAse (Roche Diagnostics) for 1 h at 37 °C under shaking. The cell suspension was then filtered through a 100 μm nylon mesh and washed by 2 further centrifugation steps in DMEM. Pellets were resuspended in Colorectal Cancer Spheroid Cells (CCSCs) medium [16] supplemented with 10 mM nicotinamide, 1 mM Y-27632 (both from Sigma-Aldrich), 20 ng/ml human EGF and 10 ng/ml human basic fibroblast growth factor (both from PeproTech). The resulting suspension was plated in ultra-low attachment tissue culture flasks (Corning Costar), and cultured in humidified atmosphere at 37 °C, 5% CO_2_. Every 2 to 3 days, half of the culture medium was refreshed. Clusters of proliferating cells became evident after a variable length of time, ranging from 5 days to 3 weeks. Cultures in which no proliferating clusters were detected after 4 weeks were discarded. The resulting multicellular spheroid cultures were then passaged weekly and used for in vitro and in vivo experiments within the 12th passage. Genomic DNA was routinely extracted from CCSCs and patient-matched nontumoral tissues with the Dnasy Mini Kit (Qiagen) and used for mutation analysis [29] and for Short Tandem Repeats (STR) analysis. The latter was performed with the AmpFlSTRIdentifiler Plus Kit (Applied Biosystems) and used to generate a unique STR profile for each primary CRC cell line, which was used to monitor purity of the line over time and to confirm its matching with the original patient material. CCSCs were then routinely tested for their ability to produce colon adenocarcinomas histologically identical to the human tumors of origin when injected into NOD.Cg-Prkdc^scid^ Il2rg^tm1Wjl^/SzJ (NSG) mice (The Jackson Laboratory) as previously described [29]. Primary CRC cells used in this study were obtained from a 63 years old male CRC patient undergoing surgery for G3 TNM IIIC right colon tumor and displayed mutated *APC*, *TP53*, *PI3KCA* and *KRAS* and from a 65 years old female CRC patient undergoing surgery for G2 TNM IIA right colon tumor with mutated *APC* and wild-type *KRAS*, *TP53*, *PI3KCA*. SW480 cells were purchased from the American Type Culture Collection (ATCC) and cultured in DMEM supplemented with 10% heat-inactivated fetal bovine serum, 100 U/ml penicillin, and 10 μg/ml streptomycin (Thermo Fisher Scientific) at 37 °C in a 5% CO_2_ atmosphere. Cultured cells (both primary and commercial lines) were routinely tested for mycoplasma contamination with the PCR Mycoplasma Test Kit (PanReac AppliChem).

### Antibodies and reagents

Monoclonal antibodies against PROMININ-1 (AC133 epitope both pure #130–090-423 used for immunofluorescence and biotinylated #130–090-664 used for flow cytometry, 1:10) were obtained from Miltenyi Biotec. Monoclonal anti-Ki67 (Dako, Agilent Technologies, #M7240, 1:200) and polyclonal anti-Ki67 (Santa Cruz Biotechnology, #sc-15,402, 1:200) were used for immunofluorescence. EpCAM-APC used for flow cytometry (#347200, 1:40) was from Becton Dickinson. Monoclonal anti-ZEB2 (#sc-271,984, 1:200) used for immunofluorescence was from Santa Cruz Biotechnology. Anti-CRAF pS338 (MA1–90087, 1:100) used for immunofluorescence was from Thermo Fisher and anti-CRAF pS338 (#56A6, 1:1000) used for western blot was from Cell Signaling Technology. Anti-ASK1 pS83 (#3761, 1:1000), VIMENTIN (#5741), CADHERIN-2 (#13116), SNAI1 (#3879), SNAI2 (#9585), TCF8/ZEB1 (#3396) used for western blot were from Cell Signaling Technology, while anti-CADHERIN-1 (#610181) was from Becton Dickinson. Monoclonal anti-β-ACTIN (#A5316, 1:10000) used for western blot was from Sigma-Aldrich. Secondary mouse IgG, HRP-linked (#NA931, 1:4000) and rabbit IgG, HRP-linked (#NA934V, 1:4000) were from GE Healthcare Life Sciences. Secondary antibodies, goat anti-mouse IgG Alexa Fluor®647-conjugated (#A21235, 1:1000), goat anti-rabbit IgG Alexa Fluor®555-conjugated (#A21428, 1:1000), streptavidin 647 (S32357, 1:250), and 4′,6-diamidino-2-fenilindole (DAPI, #D1306, 100 nM) were obtained from Thermo Fisher Scientific. PKH26 (PKH26GL, Sigma-Aldrich) for cell membrane labeling was used diluted 1:1000 and cells were stained following manufacturer’s instructions. ProLong Gold Antifade (#P7481) was from Thermo Fisher Scientific. Mayer’s haematoxylin (#MHS32) and Eosin (#HT110232) were from Sigma-Aldrich and used according to the manufacturer’s protocol. Etoposide (#E1383) and irinotecan (#I1406) were from Sigma-Aldrich, oxaliplatin and 5-fluorouracil were from Peviva. Agarose (SeaPlaque GTG agarose, #50111) was from Lonza. Crystal violet (#C3886) was from Sigma-Aldrich and used 0.1% in 10% MetOH. Triton X-100 (#1610407) was from Bio-Rad Laboratories and used at 0.1%. Stripping buffer was from Thermo Fisher Scientific (#21059) and used according to the manufacturer’s protocol. Matrigel (Corning® Matrigel® Growth Factor Reduced (GFR) Basement Membrane Matrix) was purcheased from Corning (#354230).

### Animal procedures

All animal procedures were performed according to the Italian National animal experimentation guidelines (D.L.116/92) upon approval of the experimental protocol by the Italian Ministry of Health’s Animal Experimentation Committee (DM n. 292/2015 PR 23/4/2015). 6-week-old female NOD-SCID mice from Charles River Laboratories were used for PKH26 experiments and 6-week-old female NOD.Cg-Prkdc^scid^ Il2rg^tm1Wjl^/SzJ (NSG) mice (The Jackson Laboratory) were used for exogenous ZEB2 expression experiments. For PKH26 experiments, 5 × 10^5^ CCSCs were injected subcutaneously in the flank of NOD/SCID mice, in 100 μl 1:1 PBS/Matrigel (BD Biosciences). Tumors were measured twice weekly by an external digital caliper, and volumes were calculated using the following formula: π/6 x d2 x D, where d and D represent shorter and longer tumor measurements, respectively. Mice were grouped and sacrificed at different time point (1, 3, 6 weeks after injection) for subsequent studies. For exogenous *ZEB2* expression experiments, 10^4^ CCSCs or SW480 cells transduced with pLenti-GFP and pLenti GFP-ZEB2 were injected subcutaneously in the flank of NSG mice as described above. Drug treatments started when tumor volume reached 50–100 mm^3^. Mice were randomized in control and treatment group and treated with 12,5 mg/kg 5-fluorouracil and 5 mg/kg oxaliplatin intraperitoneally weekly. Control animals were treated with vehicle only. Tumor growth was measured at the indicated time points. Animals were euthanized according to the national Animal Welfare Guidelines.

### Reverse-phase protein Array

Following FACS separation, CCSCs were promptly lysed in 10 μl extraction buffer [50% 2X Tris-Glycine SDS Sample Buffer (Life Technologies), 47.5% 1X with T-PER reagent (Thermo Fisher Scientific and 2.5% Tris (2-carboxyethyl) phosphine hydrochloride (TCEP) reagent (Thermo Fisher Scientific)]. Lysates were boiled for 3 min and stored at − 80 °C until further processing. Prior to printing on nitrocellulose slides (GRACE Bio-Labs Inc.) via a robotic arrayer (Aushon Biosystems), samples were thawed and boiled 3 min. In order to increase the amount of protein deposited on each slide, printing was performed by using 5 depositions per spot and samples were printed in technical triplicates. Reference standard lysates, i.e. HeLa + Pervanadate (Becton, Dickinson and Company), A431 + EGF (Becton, Dickinson and Company), Jurkat + Etoposide (Cell Signaling Technology) and Jurkat + Calyculin A (Cell Signaling Technology), were printed in 10-point decreasing mixtures of treated to untreated samples as procedural controls and as positive controls for antibody staining. Each reference standard curve was printed in technical triplicate at a final concentration of 0.5 mg/ml. A selected subset of the printed microarray slides were stained with Sypro Ruby Protein Blot Stain (Thermo Fisher Scientific) to estimate sample total protein concentration and the remaining slides were stored under desiccated conditions at − 20 °C. Immediately before antibody staining, printed slides were treated with 1X Reblot Mild Solution (Chemicon) for 15 min, washed 2 × 5 min with PBS and incubated for 2 h in blocking solution containing 2% I-Block (Applied Biosystems) and 0.1% Tween-20 in PBS. Immunostaining was carried out using a tyramide-biotin signal amplification kit (DAKO). Primary antibody binding was detected using a biotinylated goat anti-rabbit IgG H + L (diluted at 1:7500; Vector Laboratories) or rabbit anti-mouse Ig (diluted at 1:10, DAKO) followed by streptavidin-conjugated IRDye®-680LT fluorophore (LI-COR Biosciences). Primary antibodies underwent pre- and post-RPPA validation for single band specificity by western blot using complex cellular lysates. Negative control slides, incubated only with secondary antibody were included in each staining run. All Sypro Ruby and immunostained slides were scanned using a Tecan Power Scanner™ (Tecan Group Ltd) at 5 μm resolution. Acquired images were analyzed with MicroVigene v5.2 (VigeneTech) for spot detection, local and negative control background subtraction, replicate averaging and total protein normalization. The “R” software packages ‘reshape2’, ‘ggplot2’, ‘coin’, ‘gplots’ and ‘shiny’ were used to carry out slide quality control, internal standardization, two-way hierarchical clustering (Euclidean distance and Ward.D2 method), Kruskal-Wallis and Wilcoxon Rank Sum non-parametric statistical tests (Benjamini & Hochberg criterion was used for multiple comparisons adjustment with an accepted false discovery rate of 0.05). A detailed list of antibodies used for RPPA is available in Additional file 1: Table S1.

### Real-time PCR

Total RNA was extracted with TRIzol (Thermo Fisher Scientific) following manufacturer’s instructions. 1 μg of RNA was reverse transcribed with M-MLV reverse transcriptase (Thermo Fisher Scientific) and 50 ng of cDNA were used as template in the PCR reactions. Specific probes used for *ZEB2, MKI67, BMI1, β-ACTIN* and *NANOG* were all from Thermo Fisher Scientific (Additional file 2: Table S2) and specific primers for *ZEB1, CDH1, VIMENTIN, SNAI1, SNAI2, CDKN1B,* (Additional file 3: Table S3) were from Sigma-Aldrich. Normalization was performed using *β-ACTIN* as reference. RNA from xenografts derived from pLenti-GFP and pLenti GFP-ZEB2-transduced cells was extracted and reverse transcribed as described above. To analyze the expression of cell cycle-associated genes TaqMan® Array, Human Cyclins & Cell Cycle Regulation, Fast 96-well (Thermo Fisher Scientific) was used following the manufacturer’s instructions. Values were expressed in terms of 2^-ΔΔCt^ where ΔΔCT = ΔCTsample−ΔCTcalibrator or ΔCt. ΔCt is the difference in threshold cycles between the specific RNA and reference gene amplicons given by StepOne Plus Real-Time PCR software by negative correlation with an internal reference dye (ROX).

### Human transcriptome array

PKH26^+^ and PKH26^−^ xenograft-derived CCSCs were FACS-sorted as described in the flow cytometry section and processed with the HTA 2.0 Affymetrix array following the manufacturer’s instructions. The data matrix having as rows (statistical units) and as columns (variables) of the 10 samples (5 PKH26^+^ and 5 PKH26^−^) was analysed by means of Principal Component Analysis (PCA) to single out an independent component allowing for the complete partition of PKH26^+^ and PKH26^−^ samples in the loading space [30]. The transcripts having the highest absolute score in the discriminant component were identified. The replicated entries of genes (for PKH26^−^ samples: *Homo sapiens* piRNA piR-43,853 complete sequence, transfer RNA Gly (anticodon TCC), transfer RNA Ile (anticodon AAT), transfer RNA Leu (anticodon AAG), transfer RNA Leu (anticodon TAG), transfer RNA Pro (anticodon AGG), transfer RNA Pro (anticodon CGG); for PKH26^+^ samples: *Homo sapiens* piRNA piR-31,233 complete sequence, *Homo sapiens* piRNA piR-35,626 complete sequence, *Homo sapiens* piRNA piR-37,799 complete sequence, *Homo sapiens* piRNA piR-38,408 complete sequence, *Homo sapiens* piRNA piR-53,527 complete sequence, *Homo sapiens* piRNA piR-55,000 complete sequence, *Homo sapiens* piRNA piR-57,434 complete sequence) were both selected as conditions-related genes allow for a quality proof of the results.

### PKH26 staining

SW480 or CCSCs (the latter previously dissociated with TrypLE Express, Thermo Fisher Scientific) were stained for 2 min at 37 °C with PKH26 (Sigma), then washed extensively with PBS. PKH26 staining was assessed by flow cytometry and cells were used for subsequent experiments only when positivity was ≥98%. For in vivo experiments 5 × 10^5^ PKH26-stained cells were injected subcutaneously in NSG mice, which were sacrificed at different times for the detection of PKH26^+^ cells or at 3 weeks post-injection for all the other experiments.

### Lentiviral infection

CCSCs or SW480 cells were stably transduced with pLenti-GFP (lentiviral vector with C-terminal GFP tag, catalogue number PS100065) or pLenti GFP-ZEB2 (catalogue number RC215227L2) purchased from Origene (Rockville, MD, USA).

### Flow cytometry, cell cycle analysis and cell sorting

For flow cytometry experiments, xenografts derived from PKH26-stained cells were cut into small pieces, washed with ice-cold PBS, and subsequently digested with TrypLE express for 15 min at 37 °C with vigorous pipetting every 5 min. Freshly isolated cells were stained with biotinylated anti-PROMININ-1 and anti-EpCAM and specific secondary antibodies. 10 μg/ml 7-aminoactinomycin D was used for dead cell exclusion. The cell cycle status of CCSCs and SW480 xenograft cells transduced with the pLenti-GFP vector or with pLenti-GFP-ZEB2 was assessed by staining dissociated cells with 50 μg/ml propidium iodide dissolved in buffer 0.1% trisodium citrate, 9.65 mM NaCl, 0.1% Nonidet P40, and 200 μg /ml RNase for 1 h at room temperature. Samples were analyzed with a FACSCanto flow cytometer (Becton Dickinson) equipped with a DIVA software. To obtain EpCAM^+^/PKH26^+^ and EpCAM^+^/PKH26^−^ or pLenti-GFP and pLenti GFP-ZEB2 fractions, cells were sorted with a FACSAria (Becton Dickinson).

### Immunofluorescence

CCSCs were centrifuged at low speed on polylysine-coated glass slides, whereas SW480 cells were grown directly on glass slides. Cells were then fixed in 2% paraformaldehyde (PFA) for 15 min at room temperature and permeabilized in 0.1% Triton X-100 for 5 min at RT then, after two washes in PBS, they were incubated with glycine 1 M (Sigma-Aldrich) 1 h at room temperature. Glycine was removed without washing and, after blocking in 3% BSA (Sigma-Aldrich)/3% FBS (Gibco)/PBS (Sigma-Aldrich), cells were incubated overnight at 4 °C with primary antibodies anti-Ki67, CRAF pS338 and anti-ZEB2. After two washes in PBS, cells were incubated with appropriate secondary antibodies in a buffer containing DAPI, 3% BSA, 5 μg/ml RNAse (Roche) diluited in PBS for 1 h at room temperature in the dark. Subsequently, glasses were mounted with ProLong Gold Antifade. Immunofluorescence staining of xenograft-derived sections was performed as follows: sections were fixed in 2% PFA for 15 min at room temperature, washed two times in PBS and permeabilized in 0.1% Triton X-100 for 5 min at room temperature then incubated overnight at 4 °C with primary antibodies anti CRAF pS338, anti PROMININ-1, anti Ki67 and anti ZEB2. After washing in PBS, sections were incubated with a mixture of appropriate secondary antibodies and DAPI as described above. SW480 cells were seeded 5 × 10^4^ cells/ml and treated after 24 h with etoposide 10 μM or irinotecan 10 μM for 48 h. Cells were processed for immunofluorescence as described above and stained with anti-pCRAF, the appropriate secondary antibody and DAPI for nuclear identification. Slides were analyzed at room temperature on a FV-1000 confocal microscope (Olympus) equipped with Ultraplan Apochromatic 60X N.A.1.42 and 40X N.A. 1.30 oil immersion objectives and acquired with the Olympus Fluoview software. The resulting images were not subjected to further processing.

### Western blotting

Cultured cells or ~ 50 mg pieces of frozen xenografts were lysed in the appropriate volume of the respective lysis buffer: for cultured cells we used 1% NP40 lysis buffer (20 mM Tris HCl pH 7.2, 200 mM NaCl, 1% NP40), while for xenograft tissues we used 10 mM Tris pH8, 150 mM NaCl, 60 mM Octyl-β-Glucoside. Both buffers were supplemented with protease inhibitor cocktail and phosphatase inhibitor cocktails I and II (all from Sigma-Aldrich). Tissues were homogenized with Pro 200 Kema Keur (Pro Scientific Inc. Oxford) at maximum speed at 4 °C for 30 s. Lysate concentration was determined with the Bradford assay (Bio-Rad Laboratories) and equal amounts of proteins were loaded on a 4–12% precast gel (Thermo Fisher Scientific) and transferred to nitrocellulose membranes (GE Healthcare Life sciences). Blots were blocked with TBST 5% nonfat dry milk (Bio-Rad Laboratories) and incubated overnight at 4 °C with primary antibodies diluted in TBST/BSA 5%, after 4 washes in TBST then incubated for 45 min with specific secondary HRP-conjugated antibodies diluted in TBST 5% nonfat dry milk. Images were taken and analyzed with Bio-Rad ChemiDoc Imagers (Bio-Rad Laboratories). For densitometry quantification immunoblot signals were acquired with ChemiDocMP (BioRad) and the relative intensity was quantified with Image Lab software. Normalization was performed using β-ACTIN as reference.

### RNA interference

1.25 × 10^5^ CCSCs or SW480 cells were seeded on six-well plates in antibiotic-free culture medium and incubated for 4 h at 37 °C in the presence of 320 nM ON-TARGETplus SMARTpool non-targeting siRNA (D-001810-01-055), human ZEB2 siRNA (L-006914-02-0005) (Dharmacon/Thermo Scientific) and 5 μl Lipofectamine 2000 (thermo fisher scientific). After 4 h the transfection mixture was substituted with the appropriate culture medium and cells were analyzed for cell viability, gene and protein expression at the indicated times

### Cell viability assay

The viability of CCSCs or SW480 cells was determined by CellTiter-Glo luminescent cell viability assay (Promega) according to the manufacturer’s directions. CCSCs and SW480 cells (2.5 × 10^3^ per well) were seeded in 96-well plates (three replicates per experimental point) in the appropriate medium and incubated in a humidified atmosphere at 37 °C, 5% CO_2_. For in vitro chemotherapy treatment, cells were treated for 48 h with 10 μM 5-fluorouracil or 10 μM oxaliplatin. Luminescence was detected with a DTX880 multimode microplate reader (Beckman Coulter).

### Statistical analyses

Statistical analyses were performed using GraphPad Prism version 4.0 for Windows (GraphPad Software) with non-paired double-tailed t test (after verifying normal distribution of the population with Shapiro–Wilk test) or with one-way ANOVA where appropriate. Results are presented as the mean ± SD or mean ± SEM where appropriate. Statistical significance is expressed as *, *P* < 0.05, **, *P* < 0.01 and ***, *P* < 0.001. Statistical analysis of Affymetrix results and of Reverse Phase Proteomic Arrays (RPPA) are described in the specific Supporting Methods sections and/or in the respective figure legends. Principal Component Analysis was performed by SAS version 8.1.

## Results

### Isolation and characterization of QCSCs responsible for chemoresistance in colorectal tumors

**Fig. 1:**
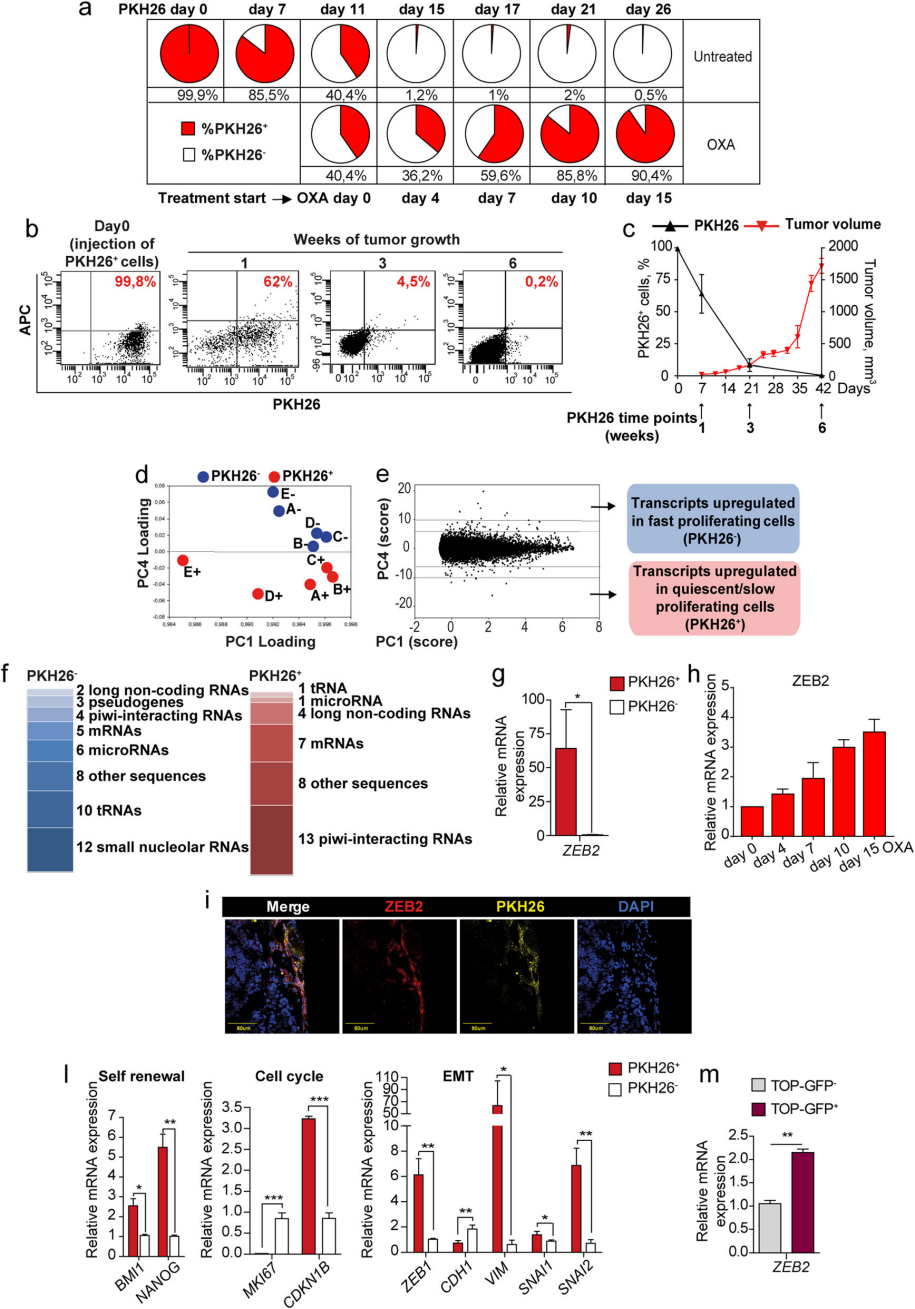
Untreated tumors contain chemotherapy-resistant quiescent cells with an EMT/stemness phenotype and increased ZEB2 levels. **a** SW480 cells were stained with PKH26, treated with 2,5 μM oxaliplatin (OXA) at day 11 and monitored by flow cytometry. FACS plots are shown in Additional file 4. **b** FACS plots showing PKH26 positivity in CCSCs-derived tumors the day of injection (Day 0) and at 1/3/6 weeks. **c** Percentage of PKH26^+^ cells (black line) in relation to tumor size (red line). Mean ± SD or SEM (tumors), *n* = 6 tumors/group. **d** Spatial representation of principal component (PC) analysis with genes as rows (statistical units) and samples as columns (variables). *n* = 5 samples of 2 pooled tumors each. **e** Numerical PC plot identifying genes with the highest absolute score in the discriminant component. A full list of transcripts modulated in PKH26^+^ versus PKH26^−^ cells is reported in Additional file 5. **f** Categories of transcripts enriched in PKH26^+^versus PKH26^−^ cells. Replicated entries are reported in Materials and Methods. Transcripts categories are detailed in Additional file 6. **g** qRT-PCR analysis of *ZEB2* in EpCAM^+^/PKH26^+^ versus EpCAM^+^/PKH26^−^ cells from CCSCs-derived xenografts. *P < 0.05 (two-tailed t test), mean ± SD, *n* = 3 pools of 6 tumors each. **h** qRT-PCR of *ZEB2* expression in SW480 cells untreated (day 0) or treated with 2,5 μM oxaliplatin (OXA). Mean ± SD of 3 experiments. **i** Representative confocal image of CCSCs-derived xenograft sections showing overlapping areas of ZEB2 (red) and PKH26^+^ (yellow) positivity. Scale bar 80 μm. **l** qRT-PCR of xenograft-derived EpCAM^+^/PKH26^+^ versus EpCAM^+^/PKH26^−^ cells. *P < 0.05, **P < 0.01 and ***P < 0.001 (two-tailed t test). Mean ± SD, *n* = 3 pools of 6 CCSCs-derived tumors each. **m** qRT-PCR analysis of *ZEB2* expression in TOP-GFP.mcherry negative (grey) and positive (purple) CCSCs sorted from in vitro culture. Mean ± SD of 3 experiments

Seminal studies on the dynamics of chemotherapy response pointed to a rare cell population that remains latent throughout the life of untreated tumors and emerges only upon chemotherapy treatment [18]. We ought to analyze such “pre-existing persisters” by using the proliferation-sensitive dye PKH26, which incorporates into lipid membranes and is progressively lost during subsequent cell divisions. In our previous studies, we previously demonstrated that PKH^+/high^ CRC cells possessed a higher tumorigenic potential as compared with PKH^−/low^ cells, indicating that the quiescent/slow cycling fraction is enriched in cells with stemness features [16]. The PKH26 experimental system gave us the possibility to identify cells that are quiescent/slow cycling from the initial stages of tumor development and not just in a given moment (as occurs instead with the H2B-GFP system). First, we aimed to determine whether PKH26^+^ cells survived chemotherapy treatment. SW480 CRC cells were stained with PKH26, then allowed to divide for 11 days, after which chemotherapy treatment was started. We monitored the percentage of PKH26^+^ cells for two additional weeks, during which the positive population decreased to 0.5% in the original culture but increased to more than 90% in chemotherapy-treated samples (Fig. 1a). Drug-treated samples consisted of ~ 60% live cells after 2 weeks, as shown by 7-AAD staining (Additional file 4: Figure S1a). This observation indicates that cells selectively surviving chemotherapy are the same cells that were quiescent/slow cycling in untreated tumors and not cells that entered quiescence upon drug treatment. Therefore, we focused our attention on cells present in untreated tumors that are destined to survive chemotherapy treatment and we undertook their isolation and characterization. To do this, we used molecularly annotated 3D cultures of primary CRC cells (thereafter called CCSCs, Colon Cancer Spheroid Cells) that were previously shown by our group and others to faithfully reproduce original patient tumors when inoculated in immunecompromised mice [17, 29, 31]. PKH26-stained and sorted CCSCs were inoculated into the flanks of NSG mice and the percentage of PKH26^+^ cells was monitored over time by flow cytometry (Fig. 1b and c). At 3 weeks post-injection we isolated from tumor xenografts EpCAM^+^/PKH26^+^ and EpCAM^+^/PKH26^−^ cells (Additional file 4: Figure S1b) that were used for further characterizations. Flow cytometry analysis showed that PKH26^+^ xenograft cells were negative for Ki67 and expressed very high levels of PROMININ1, indicating a stem cell phenotype (Additional file 4: Figure S1c and d), in line with our previous observations [16]. In order to investigate whether long-term quiescent cells were characterized by a specific pattern of gene expression, we analyzed PKH26^+^ and PKH26^−^ cells freshly isolated from CRC tumor xenografts with the Affymetrix 2.0 human transcriptome array. The existence of a gene signature able to discriminate between the two populations was investigated through a purely unsupervised data driven approach suitable to identify small sets of biologically relevant genes in an otherwise similar background [30]. Principal component analysis (PCA) of the results showed a sharp distinction between profiles of fast proliferating and quiescent/slow proliferating cells emerging from the fourth PCA component (PC4) which, although accounting for only 0.15% of gene expression variability, nevertheless allowed for a perfect partition of the loading component space into PKH26^+^ and PKH26^−^ areas (Fig. 1d). Setting two thresholds respectively at 6 and 10 standard deviation units from the mean (Fig. 1e), we identified transcripts mostly affected by PC4 and consequently more involved into PKH26^+^/ PKH26^−^ discrimination (detailed in Additional file 5: Table S4). The great majority of transcripts differentially modulated in PKH26^+^ and PKH26^−^ cells did not correspond to structural genes but rather to post-transcriptional regulators (microRNAs, small nucleolar RNAs, piwi-interacting RNAs, long non-coding RNAs and tRNAs) (Fig. 1f and Additional file 6: Table S5), indicating that the balance between quiescence and proliferation relies on the fine tuning of a basically similar transcription pattern. Among the transcripts more expressed in QCSCs we found the long non-coding RNA relative to the transcription factor ZEB2 (zinc finger E-box binding homeobox 2), previously known for its involvement in EMT and TGF-β-regulated processes [32–34]. Moreover, the *ZEB2* mRNA had a statistically significant score on PC4 (− 2,34, *P < 0.01). Therefore, we decided to explore the expression and function of ZEB2 in CRC cells. We confirmed the enrichment of *ZEB2* mRNA in PKH26^+^ cells isolated ex vivo from tumor xenografts and in chemotherapy-treated cells (Fig. 1g and h), while in xenograft sections ZEB2-expressing areas overlapped with PKH26^+^ areas (Fig. 1i). ZEB2 expression in PKH26^+^ tumor cells was accompanied by an increased expression of CRC self-renewal factors *BMI1* and *NANOG* [35, 36], of EMT-related genes *ZEB1*, *VIMENTIN*, *SNAI1* and *SNAI2*, of cyclin-dependent kinase inhibitor 1B (*CDKN1B*, encoding for p27^Kip1^) and by lower levels of *MKI67* and *CADHERIN-1* (Fig. 1l), indicating that the QCSCs population in colorectal tumors is characterized by stemness and EMT features. In line with these observations, we analyzed CCSCs expressing the TOP-GFP vector as a functional marker of β-catenin activity and a surrogate marker of CRC stem cells [37]. Sorted CCSCs with higher levels of TOP-GFP (and consequently of β-CATENIN-dependent transcription) expressed higher levels of *ZEB2* (Fig. 1m), further supporting the stemness of quiescent/slow cycling CRC cells.

### Global pathway analysis shows the activation of chemoresistance-related factors in QCSCs

**Fig. 2:**
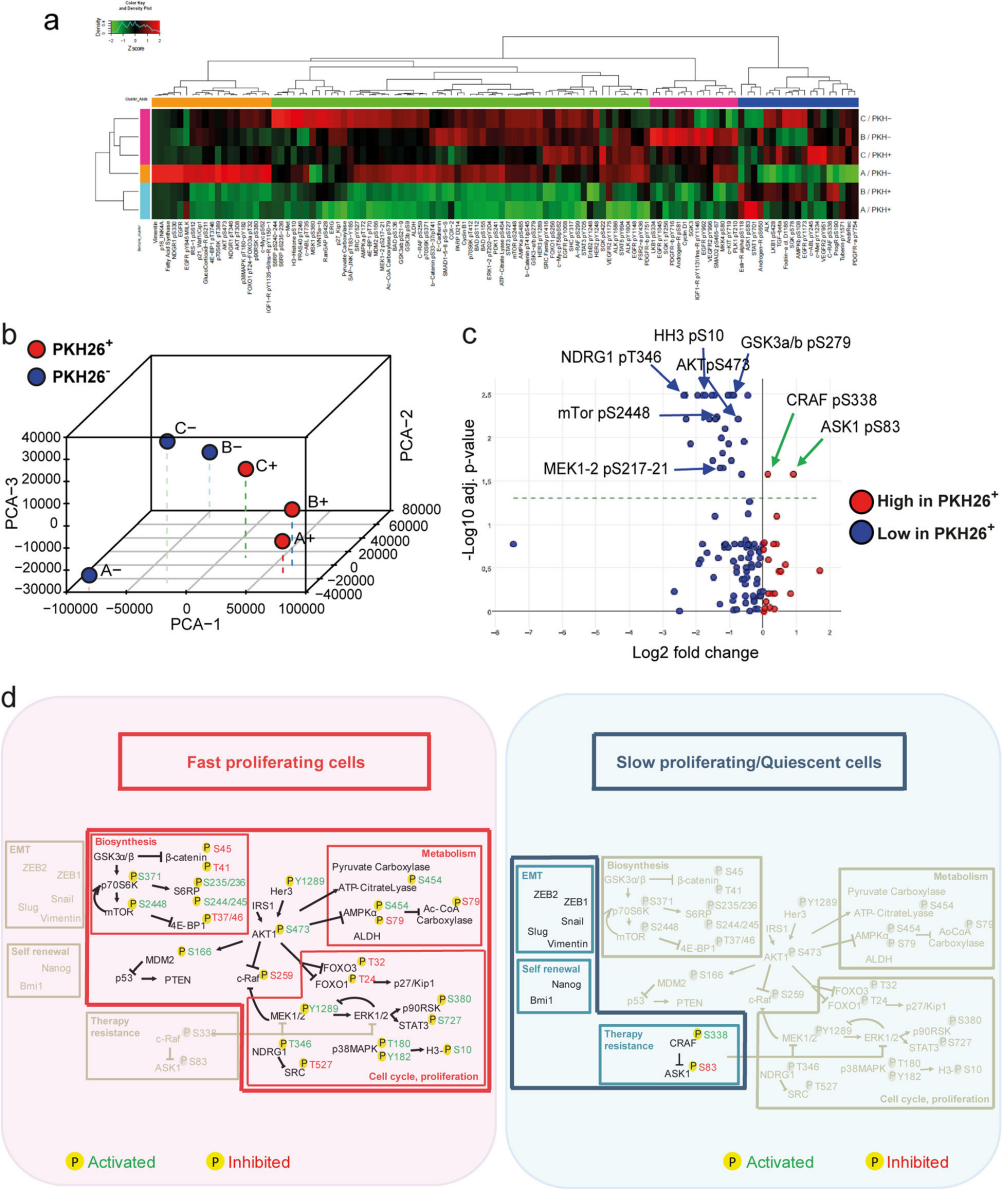
Reverse-phase proteomic analysis of quiescent/slow cycling xenograft cells. **a** Hierarchical clustering of RPPA results obtained on 3 EpCAM^+^/PKH26^+^ and EpCAM^+^/PKH26^−^ cell samples isolated from CCSCs-derived tumor xenografts. Clusters, identified for either antibodies or samples and based on optimal cut of dendrograms, are indicated by coloured bars adjacent to dendrograms. The values represented by the heatmap correspond to normalized intensities of antibodies, standardized over the sample set analyzed (z score). *n* = 3 pools of 12 tumors each. A list of RPPA antibodies and modulated endpoints are reported respectively in Additional file 1: Table S1 and Additional file 7: Table S6. **b** Principal component analysis (PCA) of RPPA results showing that PKH26^+^ samples have a common molecular signature. **c** Volcano plot showing the antilogarithm (base = 10) of the adjusted *P* value versus base 2 logarithm of the ratio between PKH26^+^ and PKH26^−^ samples. Kruskal Wallis test was performed for each RPPA analyte on the 3 samples stratified by PKH26 positivity. RPPA analytes where Kruskal Wallis test resulted in a statistically significant (*P < 0.05) change between PKH26-stratified samples, underwent a further analysis by means of Wilcoxon signed-rank test. All the resulting *p* values were adjusted for multiple comparisons using the Benjamini-Hochberg correction. **d** Schematic representation of pathways that emerged from experiments described in Figs.1 and 2 as present in PKH26-negative fast proliferating cells (left, red area) or in slow proliferating/quiescent cells (right, blue area). Phosphorylation sites are outlined in green when they result in protein activation, in red when they inhibit protein function. Activated pathways are highlighted in colors while inhibited pathways are depicted in light grey

Reverse-Phase Protein Array (RPPA) allows the simultaneous evaluation of phosphorylated, cleaved, or unmodified proteins generating comprehensive profiles of pathway activation in different cell or tissue samples [38, 39]. RPPA was used to compare PKH26^+^ and PKH26^−^ cells isolated ex vivo from CCSCs-derived tumor xenografts in order to obtain a broad picture of signaling pathways modulated in these two populations. Three matched pools of ex vivo PKH26^+^/PKH26^−^ cells were analyzed with the antibodies reported in Additional file 1: Table S1. Hierarchical clustering showed that two samples of QCSCs had a massive down modulation of most pathways, particularly those involved in proliferation and biosynthesis (Fig. 2a). The third sample of quiescent cells showed a down regulation of most pathways but a simultaneous upregulation of a small set of phosphoproteins (c-Met, VEGFR2, c-Abl, SGK) (Fig. 2a), indicating the existence of multiple layers of quiescence-associated signals. Nevertheless, principal component analysis (PCA) of RPPA results highlighted a molecular signature common to quiescent/slow proliferating CRC cells (Fig. 2b). Statistically significant endpoints modulated in PKH26^+^ and PKH26^−^ cells (shown in detail in Additional file 7: Table S6) are summarized in Fig. 2c, where QCSCs are sharply identified by increased levels of CRAF S338 phosphorylation and ASK1 S83 phosphorylation. Importantly, pS338 CRAF and pS83 ASK1 have been individually implicated in protecting cells from genotoxic insults [40–42], but they have also been shown to act in concert by forming a chemoresistance-promoting complex at mitochondria [43]. Due to the specific role of pCRAF in driving therapy resistance [41], we assessed its expression in tumor xenografts, where it overlapped with PKH26^+^ and partially with PROMININ1^+^ areas (Additional file 8: Figure S2a), and we confirmed that it is actually upregulated in drug-treated CRC cells (Additional file 8: Figure S2b). Fast proliferating PKH26^−^ cells showed, among others, an increased expression of phosphorylated Akt, MEK1/2, mTOR (and downstream effectors p70S6K and 4EBP1), GSK3, histone H3 and NDRG1 (Fig. 2c). The latter is particularly interesting as it has been reported to inhibit EMT, stemness and metastasis and is related to a favorable prognosis in CRC patients [44]. In order to rule out the possibility of chance correlations in the statistical analysis of RPPA results, we complemented data shown in Fig. 2c with a further analysis having samples as variants and protein endpoints as units. In fact, since the samples differ only for a transient functional state (proliferative status), they have a largely overlapping RPPA profile that translates into a major principal component explaining the major part (80%) of the among samples variance [45]. This implies that discrimination of the two populations can only start from the second component on ward, getting rid only of a minor proportion of variance. That said, the loading space allowed for a posteriori perfect discrimination among PKH26^+^ and PKH26^−^ samples as for Factor 2 (Additional file 8: Figure S2c), which explains only 8,5% of total variance and represents a common regulatory pathway within the same cell population. We observed a remarkable superposition between the two analyses, as the large majority of endpoints are identified as discriminants in both approaches (Additional file 8: Figure S2d). However, E-Cadherin emerges from the second approach as one of the endpoints most relevant for group discrimination, adding further significance to the hypothesis that QCSCs tend towards a mesenchymal state. In summary, our results showed that the molecular diversity among fast proliferating and quiescent/slow proliferating CRC cells concentrates around distinctive pathway profiles. Rapidly proliferating cells possess high levels of proteins involved in biosynthetic/metabolic pathways and are shifted towards an epithelial-like and chemosensitive status, while QCSCs depress pathways related to cell cycle/biosynthesis/metabolism and selectively upregulate factors involved in self renewal, chemoresistance and EMT/metastatic ability (Fig. 2d).

### ZEB2, pCRAF and pASK1 are coexpressed upon chemotherapy and coregulated in CRC cells

**Fig. 3:**
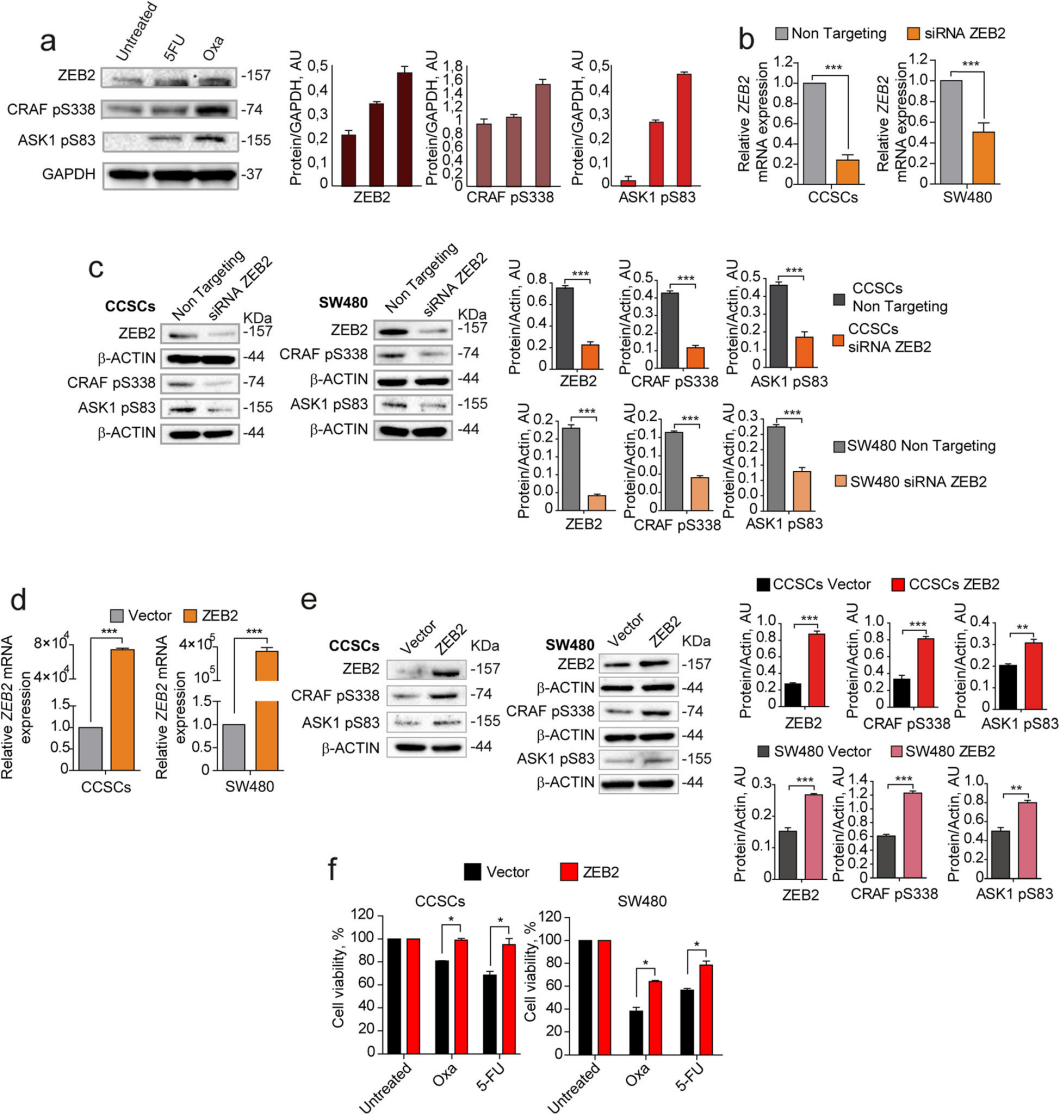
Coordinated expression and modulation of ZEB2, pCRAF and pASK1. **a** Left: immunoblot analysis of ZEB2, CRAF pS338, and ASK1 pS83 on whole lysates of SW480 cells treated for 4 days with 5 μM 5-fluorouracil (5-FU) or 2,5 μM oxaliplatin (OXA). Glyceraldehyde 3-phosphate dehydrogenase (GAPDH) was used as a loading control. Right: quantification of immunoblot shown on the left. **b** qRT-PCR analysis of *ZEB2* levels in CCSCs (left panel) and SW480 (right panel) 24 h after siRNA-mediated silencing of *ZEB2*. ***P < 0.001 from two-tailed t test. Data of qRT-PCR are the mean ± SD, *n* = 3. **c** Immunoblot analysis of ZEB2, CRAF pS338, and ASK1 pS83 on whole cell lysates 24 h upon siRNA-mediated silencing of ZEB2 in CCSCs (left panel) and SW480 (right panel). The respective quantifications are shown on the right. **d** qRT-PCR analysis of *ZEB2* levels in CCSCs (left panel) and SW480 (right panel) transduced with empty pLenti-GFP (Vector) or with pLenti-GFP-ZEB2 (ZEB2) and sorted on the basis of GFP expression. ***P < 0.001 from two-tailed t test. Data are the mean ± SD, *n* = 3. **e** Immunoblot analysis of ZEB2, CRAF pS338, and ASK1 pS83 on whole lysates of CCSCs (left panels) and SW480 cells (right panels) transduced with pLenti-GFP (Vector) or with pLenti-GFP-ZEB2 (ZEB2) and sorted as above. The respective quantifications are shown on the right. **f** Viability of CCSCs (left) and SW480 (right) transduced with pLenti-GFP or pLenti-GFP-ZEB2, sorted on the basis of GFP expression and immediately treated for 48 h with 10 μM oxaliplatin (OXA) and 10 μM 5-fluorouracil (5-FU). *P < 0.05 from two-tailed t test, *n* = 3. Data are the mean ± SD of three independent experiments

Having identified ZEB2, pCRAF and pASK1 as factors upregulated in QCSCs, we asked whether their expression was increased upon chemotherapy and modulated in a coordinated manner. First, we analyzed ZEB2, pCRAF and pASK1 expression in chemotherapy-treated cells and we observed a parallel increase of the three factors following 5-fluorouracil and oxaliplatin treatment (Fig. 3a). Then, we investigated whether the expression of pCRAF and pASK1 was mechanistically regulated by ZEB2 by modulating *ZEB2* levels with siRNA-mediated silencing or lentiviral overexpression in either CCSCs or SW480 cells and analyzing variations in pCRAF and pASK1. Transient *ZEB2* siRNA-mediated silencing (Fig. 3b) induced a decrease in the levels of S338-phosphorylated CRAF and S83-phosphorylated ASK1 (Fig. 3c), indicating that pCRAF and pASK1 lie dowstream of ZEB2 in the quiescence/chemoresistance program. Exogenous expression of a lentiviral GFP-*ZEB2* construct (Fig. 3d) increased the expression levels of pCRAF and pASK1 (Fig. 3e) and resulted in enhanced chemoresistance of both CCSCs and SW480 cells (Fig. 3f) shortly after cell transduction and sorting. At longer times of culture, however, both CCSCs and SW480 cells transduced with ZEB2 downregulated protein levels until they reached those found in untreated cultures (Additional file 9: Figure S3a), where ZEB2 expression is limited to rare Ki67-negative cells (Additional file 9: Figure S3b). These results indicate that ZEB2 controls the levels of pCRAF and pASK1 and that its levels are strictly regulated in CRC cells.

### ZEB2 expression induces tumor transition towards a slow growing chemoresistant state

**Fig. 4:**
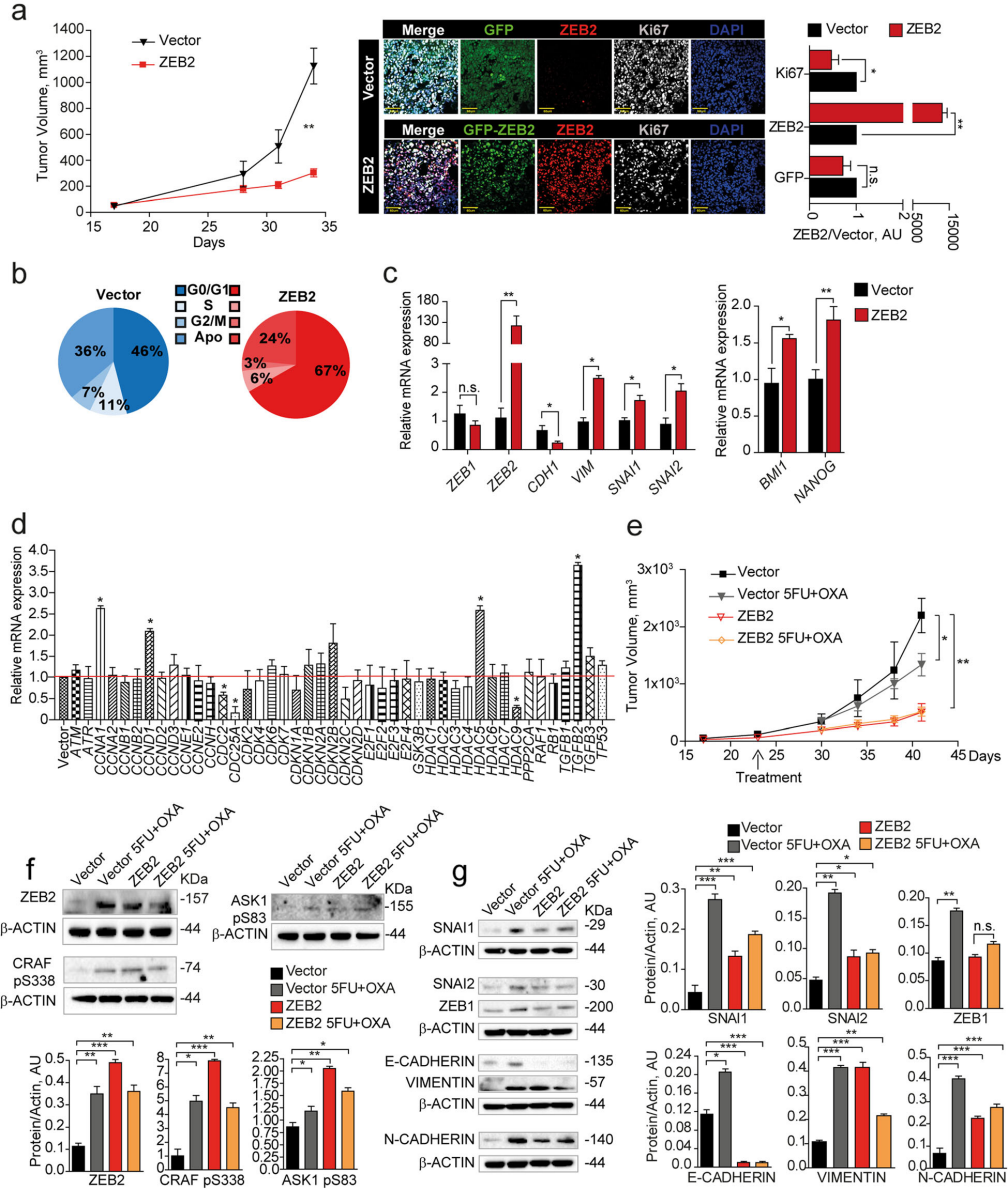
ZEB2 induces a transition towards a quiescent/slow cycling and mesechymal-like state in CRC xenografts. **a** Left: Xenograft volume of SW480 cells transduced with pLenti-GFP (Vector, black line/triangles) or with pLenti-GFP-ZEB2 (ZEB2, red line/squares). Mean ± SEM, 6 tumors/group. **P < 0.01 (two-tailed t test). Middle: representative confocal images of Vector- and GFP-ZEB2-transduced SW480 xenografts stained with anti-ZEB2 (red) and anti-Ki67 (white) antibodies. Scale bar 60 μm. Right: quantification of Ki67-, ZEB2- and GFP-positive cells performed on 3 sets composed of 5 fields/group. *P < 0.05 and **P < 0.01. Mean ± SD (two-tailed t test, n.s. = not significant). AU, arbitrary units. **b** Cell cycle analysis of GFP^+^ cells from Vector- and ZEB2-transduced tumors. **c** qRT-PCR analysis of GFP^+^ cells from Vector- and ZEB2-transduced tumors, *n* = 3 pools of 2 tumor each. *P < 0.05 and **P < 0.01 (two-tailed t test). Mean ± SD. **d** mRNA levels of cell cycle genes in GFP^+^ cells from Vector- and GFP-ZEB2-transduced tumors. Mean ± SD, *n* = 3. *P < 0.05 (two-tailed t test). **e** Volume of xenografts expressing pLenti-GFP (Vector, black line) or GFP-ZEB2 (ZEB2, red line), untreated or treated (Vector, gray line/triangles and ZEB2 yellow line/diamonds) with 5-fluorouracil plus oxaliplatin (5FU + OXA). Mean ± SEM, 6 tumors per group. *P < 0.05 and **P < 0.01 from one-way ANOVA and Bonferroni post-tests. **f** Upper panels: immunoblot analysis of ZEB2, CRAF pS338 and ASK1 pS83 on whole tumor lysates derived from SW480 xenografts. Lower panels: densitometry analysis of western blots, *n* = 3, *P < 0.05, **P < 0.01 and ***P < 0.001 (two-tailed t test). **g** Left: immunoblot analysis of EMT-related proteins on whole xenograft lysates. Every sample is a pool of 2 tumors. Right: densitometry analysis, *n* = 3, *P < 0.05, **P < 0.01 and ***P < 0.001 (two-tailed t test)

To investigate the effects of ZEB2 overexpression in vivo we inoculated freshly sorted ZEB2-transduced SW480 cells in the flanks of immunecompromised mice and analyzed xenograft growth, cell cycle status and expression of cell cycle-, EMT- and stemness-related genes. ZEB2-overexpressing tumors grew significantly slower than vector-transduced tumors (Fig. 4a, left panel) and displayed higher ZEB2 and lower Ki67 levels as compared to vector-transduced controls (Fig. 4a, right panel). Ex vivo cell cycle analysis showed that ZEB2-overexpressing tumors contained an increased proportion of cells in the G0/G1 phase of the cell cycle and a lower proportion of cells in G2/M (Fig. 4b). Assessment of EMT and self-renewal factors as determined by qRT-PCR showed that ZEB2-overexpressing tumors had increased levels of *ZEB2* itself (but not *ZEB1*), *VIMENTIN*, *SNAI1* and *SNAI2*, decreased levels of *CADHERIN1* and increased expression of *BMI1* and *NANOG* (Fig. 4c). Similar results were obtained with CCSCs, with the difference that *ZEB2*-transduced-tumors had a delayed appearance as compared to vector-transduced tumors (Additional file 10: Figure S4a-d). ZEB2-overexpressing tumors showed a modulation of several cell cycle-related factors including *CYCLINA1*, *CYCLIND1*, *CDC2*, *CDC25A*, *HDAC9* and *HDAC5* and, importantly, a strong upregulation of *TGFB2* (Fig. 4d), in line with previous studies showing a specific role of TGFβ2 in dictating the dormancy of disseminated tumor cells [46]. Then, we investigated the expression of ZEB2/pCRAF/pASK1 in vivo upon chemotherapy treatment. Vector- and ZEB2-transduced SW480 cells were inoculated into NSG mice and the resulting tumors were treated with oxaliplatin plus 5-fluorouracil for 3 weeks. In vector-transduced tumors, chemotherapy induced a growth slowdown associated to a strong increase of ZEB2, pCRAF and pASK1. Chemotherapy-treated control tumors showed also a transition towards a hybrid epithelial-mesenchymal state, as showed by the increased expression of SNAI1–2, ZEB1, VIMENTIN and N-CADHERIN but concomitant high expression of E-Cadherin (Fig. 4e-g). ZEB2-overexpressing tumors grew more slowly than controls and had a baseline higher expression of pCRAF, pASK1 and EMT-related factors with decrease of E-CADHERIN, indicating a complete EMT (Fig. 4e-g). In line with these observations, ZEB2-overexpressing tumors were unaffected by chemotherapy treatment and did not change either their slow growing rate or the expression of EMT/chemoresistance factors upon drug exposure (Fig. 4e-g). Altogether, these results identify ZEB2/pCRAF/pASK1 as factors present in rare quiescent cells in untreated tumors that are largely expressed upon chemotherapy treatment, thus inducing tumor transition towards an EMT/chemotherapy unresponsive state.

### ZEB2 expression correlates with worse prognosis and CMS4 in CRC patients

**Fig. 5:**
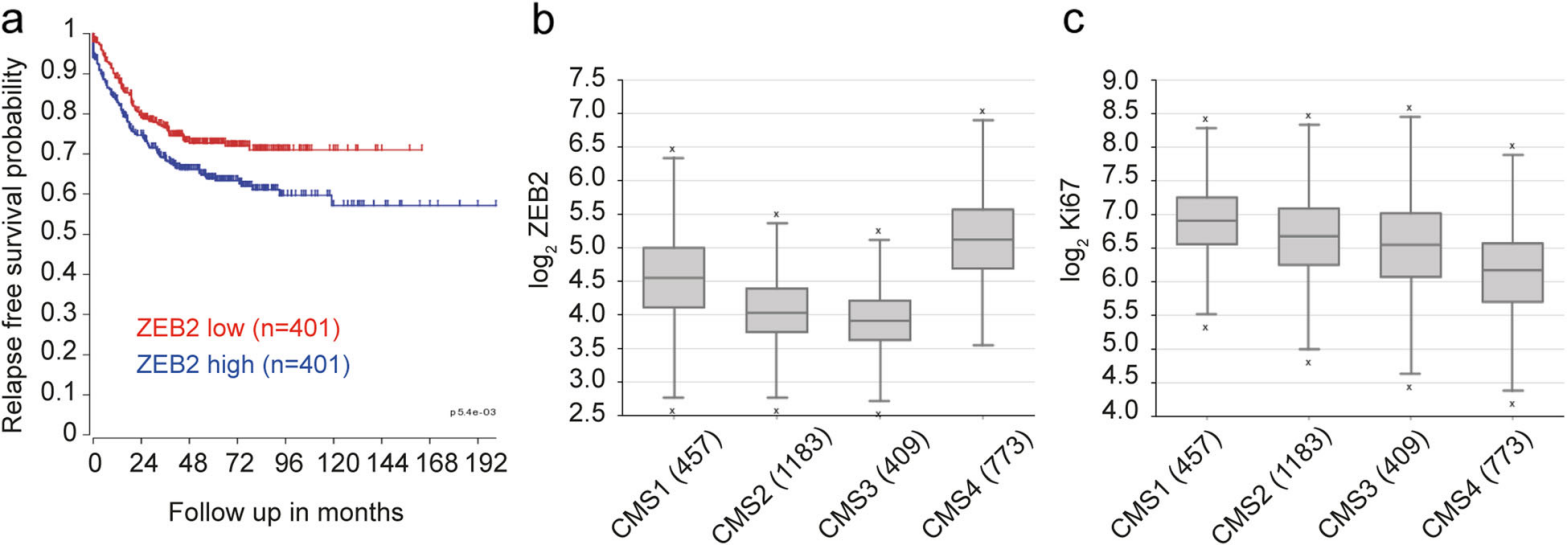
Higher ZEB2 expression is linked to CMS4 and poor prognosis in colorectal tumors. **a** Kaplan Meier curve showing the relapse-free survival of 802 CRC patients separated on the basis of ZEB2 expression (red, low expression and blue, high expression). ***P < 0.001 based on log-rank test. **b** *ZEB2* levels in CMS4 tumors as compared with CMSs 1–3. ***P < 0.001 based on one-way ANOVA, *n* = 2822. Outliers are depicted as crosses. **c** *MKI67* levels in CMS4 as compared with CMSs 1–3. ***P < 0.001 based on one-way ANOVA, *n* = 2822. Outliers are depicted as crosses. Both the analysis of variance and the single *post-hoc* pairwise comparison in **b** and **c** are highly significant

Finally, we explored the potential clinical relevance of our findings by analyzing *ZEB2* expression in a CRC dataset composed of all fresh frozen tumor samples compiled by the consensus molecular classification consortium [47]. This set, for which consensus molecular subtype (CMS) classification and in most cases stage is available, was separated by TNM stage and analyzed for *ZEB2* expression, revealing a slight but non-significant increase with progressing stage (Additional file 11: Figure S5). However, segregation of the patients into low and high *ZEB2* expression revealed a very significant increase in recurrence rate in patients with high *ZEB2* expression across all TNM stages (*p* < 0.001, *n* = 802) (Fig. 5a). Importantly, the majority of the patients in our dataset could be faithfully assigned to one of the four CMSs, which have distinguishing molecular, biological and clinical features [47]. Among these, CMS4 is typified by high expression of mesenchymal genes, prominent TGF-β activation, stromal infiltration and worse relapse-free survival [47]. In agreement with our hypothesis that ZEB2 drives an EMT-related and therapy-resistant CRC phenotype we found a significantly increased expression of *ZEB2* in CMS4 (***P < 0.001, *n* = 2822) (Fig. 5b). Likewise, consistent with the association of ZEB2 with a quiescent/slowly proliferating state, *MKI67* expression was reduced in CMS4 as compared to the other CMSs (***P < 0.001, *n* = 2822) (Fig. 5c).

## Discussion

Increasing evidence indicates that a quiescent state is tightly linked to drug resistance in cancer cells. However, due to their rareness and plasticity, quiescent cancer cells remain mostly elusive and represent a challenging therapeutic target [15]. We previously demonstrated that stem cells in CRC can be found both in the fast proliferating (PKH26^−/low^) and in the quiescent/slow proliferating (PKH26^+/high^) fraction, but PKH26^high^ cells are endowed with a higher tumorigenic potential [16]. Now, we demonstrate that quiescent/slow cycling cells present in untreated CRC xenografts are the same cells that resist chemotherapy treatment. Quiescent/slow cycling cells isolated from untreated CRC xenografts were characterized by combined features of stemness, chemoresistance and EMT, indicating that quiescence arises as a whole set of molecular traits covering multiple cellular processes. In fact, the connection between stemness and EMT was known since early studies by Mani et al., who demonstrated that normal and neoplastic mammary cells that underwent EMT exhibit stem cell markers and functional characteristics [48]. However, further studies also highlighted the implication of quiescence as a feature of CSCs undergoing EMT. To cite a few, in breast cancer Lawson and coworkers identified a metastatic cell population characterized by the expression of stem cell-, EMT-, pro-survival-, and dormancy-associated genes [9], while in acute myeloid leukemia Ebinger et al. isolated a subset of dormant stem cells with reversible properties of quiescence and therapy resistance [5]. In CRC, we found that PKH26^+^/ZEB2^+^ cells were characterized by high levels of PROMININ1, by an increased expression of self-renewal factors BMI1 and NANOG and by elevated nuclear β-CATENIN (as detected with the TOP-GFP assay), indicative of enhanced stem cell properties. Notably, ZEB2 overexpression in vivo was able to recreate a QCSCs population with features of chemoresistance and EMT. Such phenotype was almost identical to that developed by chemotherapy-treated xenografts, with the difference that ZEB2^+^ tumors appeared to have a more complete EMT (N-CADHERIN^high^/E-CADHERIN^low^) as compared to chemotherapy-treated tumors (N-CADHERIN^high^/E-CADHERIN^high^). However, different EMT states are not surprising as they are typical of CSCs populations with enhanced plasticity [15, 49]. Altogether, these observations suggest the existence of a slow cycling/mesenchymal/stem population across different tumors which may share, at least in part, a common molecular signature. Therefore, exploring the molecular features of the dormant/stem population will be particularly relevant for the identification of pharmacological strategies aimed at eradicating chemoresistant cells or alternatively at preventing their reactivation. Drugs potentially able to target dormant tumor cells may be directed against factors that play a role in both EMT and quiescence, such as those implicated in TGFβ signaling [50]. Among these, TGFβ2 was identified as crucial for the induction of dormancy in disseminated tumor cells [46] and emerged as highly upregulated in ZEB2-overexpressing tumors. These findings are also in line with the observation that *ZEB2* is highly expressed in CMS4 tumors, which are also characterized by prevalent TGFβ activation [47]. A comprehensive characterization of pathways modulated in quiescent CRC cells performed by RPPA showed a downregulation of main proliferative/biosynthetic/metabolic pathways together with an upregulation of chemoresistance factors CRAF pS338 and ASK1 pS83. Accordingly, CRAF phosphorylation in S338 was recently demonstrated to trigger a kinase-independent mechanism of DNA repair and therapeutic resistance [41]. Further underlining the tight connection between dormancy and chemoresistance, both ZEB2-overexpressing and chemotherapy-treated tumor xenografts acquired increased pCRAF and pASK expression, suggesting that this may represent a common stem in the transition through an EMT/chemoresistant state. The finding that ZEB2 is increased in CMS4 is in line with a recent study reporting that this subtype is characterized by methylation of miR200 promoter regions and consequent increased expression of EMT-related genes [51]. Indeed, the increased *ZEB2* (and decreased *MKI67*) expression detected in CMS4 tumors could be influenced by the abundant stromal infiltrate characteristic of this subtype, as stromal fibroblasts can also display a ZEB1^+^/ZEB2^+^/miR200^−^/Ki67^−^ profile [52]. In fact, the interactions between tumor cells and stromal fibroblasts have been shown to play a key role in defining poor-prognosis CRC by exploiting TGFβ signaling to drive an aggressive CSC-enriched phenotype [53]. It is likely that both the stromal and the epithelial part of CMS4 tumors contribute to the establishment of an aggressive phenotype through an interplay of signals orchestrated by TGFβ, resulting in refractoriness to conventional and targeted therapies [53, 54]. This hypothesis is corroborated by recent observations showing that budding areas of the tumor, which are in close contact with the surrounding stroma, are characterized by down regulation of proliferation genes, EMT and switching to CMS4 [55].

## Conclusions

Altogether, our results point to a ZEB2/pCRAF/pASK molecular signature involved in the determination of a quiescent/slow proliferative state that identifies a subset of cells present in baseline conditions and expanded both upon drug treatment and in aggressive CRC subtypes. The identification and characterization of quiescent drug-resistant CSCs may pave the way for future therapeutic strategies aimed at neutralizing this specific population in CRC.

## Supplementary information

**Additional file 1: Table S1.** Antibodies used for RPPA analysis.

**Additional file 2: Table S2.** Probes for gene expression analysis for qRT-PCR.

**Additional file 3: Table S3.** Primers for gene expression analysis for qRT-PCR.

**Additional file 4: Figure S1.** Chemoresistance and marker expression of PKH26-positive cells. **a** Representative flow cytometry analysis of SW480 cells stained with PKH26 and treated with 2,5 μM oxaliplatin (OXA) starting from day 11, as described in Fig. 1a. Cell viability plots obtained with 7-AAD staining are shown below PKH26 plots, and the percentage of viable cells is indicated below each plot. **b** Representative flow cytometry analysis of EpCAM and PKH26 on xenograft-derived CCSCs 3 weeks upon subcutaneous injection of PKH26-stained cells in NSG mice. IgG, isotype control antibody. **c** Representative flow cytometry analysis of PKH26 and Ki67 in xenograft-derived EpCAM^+^ CCSCs at 3 weeks of tumor growth. **d** Representative flow cytometry analysis of PKH26 and PROMININ1 in xenograft-derived EpCAM^+^ CCSCs at 3 weeks of tumor growth.

**Additional file 5: Table S4.** Gene expression array of PKH26^+^ versus PKH26^−^ cells ordered according to PCA Factor 4 and transcripts modulated in quiescent/slow proliferating (PKH26^+^) and fast proliferating (PKH26^−^) cells.

**Additional file 6: Table S5.** Categories of transcripts expressed in PKH26-positive and PKH26-negative cells.

**Additional file 7: Table S6.** RPPA endpoints.

**Additional file 8: Figure S2.** Expression of pCRAF in vivo and in vitro and complementary RPPA data analysis. **a** Representative confocal microscopy images of PKH26-positive areas (yellow) in xenograft sections immunostained with anti-pCRAF S338 (green) and PROMININ1 (red). Scale bar, 80 μm. **b** Representative confocal microscopy images of SW480 cells treated for 48 h with 10 μM etoposide or 10 μM irinotecan and stained with anti-pCRAF S338 antibody. Scale bar, 20 μm. **c** Spatial representation of principal component (PC) analysis computed on a matrix having loading values of the two components, Factor 1 and Factor 2 that discriminates among PKH26^+^ and PKH26^−^ samples. Results obtained on three PKH26^+^ versus PKH26^−^ samples, *n* = 3 pools of 12 tumors each. **d** Spatial representation of scores of components representing relative RPPA antibodies values (Factor 1 and Factor 2). Results obtained on three PKH26^+^ versus PKH26^−^ samples, n = 3 pools of 12 tumors each.

**Additional file 9: Figure S3.** Trends of ZEB2 overexpression in cultured cells. **a** Percentage of GFP positivity in CCSCs (diamonds) or SW480 (circles) cells transduced with empty pLenti-GFP (Vector) or with pLenti-GFP-ZEB2 (ZEB2) as assessed by flow cytometry for 5 weeks following lentiviral transduction and sorting (day 0). Graph shows the mean ± SD of three independent experiments. **b** Representative confocal image of CCSCs and SW480 cells transduced with GFP-ZEB2 and labeled with anti-Ki67 at day 28 after sorting. Circles indicate rare ZEB2^+^ cells, which are also Ki67^−^. Scale bar, 50 μm.

**Additional file 10: Figure S4.** In vivo effects of ZEB2 overexpression in CCSCs. **a** Volume of xenografts derived from CCSCs transduced with pLenti-GFP (Vector, black line/triangles) or with pLenti-GFP-ZEB2 (ZEB2, red line/squares). Graph shows the mean ± SEM, 6 tumors/group. **P < 0.01 from two-tailed t test. **b** Left panels: representative confocal images of xenograft sections derived from tumors obtained with primary cells transduced with pLenti-GFP (Vector) and GFP-ZEB2 (ZEB2). Sections were stained with anti-Ki67 (red). Scale bar, 70 μm. Quantification (right panel) was performed on 5 fields /group. *P < 0.05 from two-tailed t test. AU, arbitrary units. **c** Cell cycle analysis of GFP^+^ cells FACS-isolated from Vector and ZEB2-transduced tumors obtained with primary cells. **d** qRT-PCR analysis of the indicated transcripts in Vector- and ZEB2-transduced tumors obtained with primary cells, *n* = 3. *P < 0.05 and **P < 0.01 from two-tailed t test. n.s. = not significant by t test. Values are the mean ± SD.

**Additional file 11: Figure S5.** ZEB2 expression in TNM stages, correlation with RFS and CMS in stage 2 CRC patients. *ZEB2* transcript levels in the indicated number of CRC patients across all TNM stages. One-way ANOVA resulted in non-significant differences between stages. Outliers are depicted as crosses. *n* = 1079.

## Data Availability

All data generated or analysed during this study are included in this published article and its supplementary information files. Patient datasets are those used in [47] and are publicly available.

## Abbreviations

7-AAD: 7-aminoactinomycin D
ASK1: Apoptosis signal-regulating kinase
BMI1: B cell-specific Moloney murine leukemia virus integration site 1
c-Abl: Cellular homolog of the Abelson murine leukemia
CCSCs: Colorectal cancer spheroid cells
CDKN1B: Cyclin-dependent kinase inhibitor 1B
CMS: Consensus molecular subtype
CRAF: Cellular rapidly accelerated fibrosarcoma
CRC: Colorectal cancer
EMT: Epithelial–mesenchymal transition
EpCAM: Epithelial cell adhesion molecule
GSK3: Glycogen synthase kinase 3
H2B-GFP: Histone 2B-green fluorescent protein
HDAC: Histone deacetylases
MKI67: Marker Of Proliferation Kiel-67
m-TOR: Mammalian target of rapamycin
NDRG1: N-Myc Downstream Regulated Gene 1
NSG: NOD/SCID gamma chain deficient
PCA: Principal component analysis
PKH26: Paul Karl Horan 26
RPPA: Reverse Phase Protein Array
TGFβ: Transforming growth factor beta
TNM: Tumor node metastasis
VEGFR2: Vascular endothelial growth factor receptor 2
ZEB2: Zinc finger E-box binding homeobox 2
