# Supplementary figures and images for: A pre-existing population of ZEB2+ quiescent cells with stemness and mesenchymal features dictate chemoresistance in colorectal cancer
Source: J Exp Clin Cancer Res. 2020 Jan 8;39:2. doi: 10.1186/s13046-019-1505-4 (PMC6947904; doi:10.1186/s13046-019-1505-4)

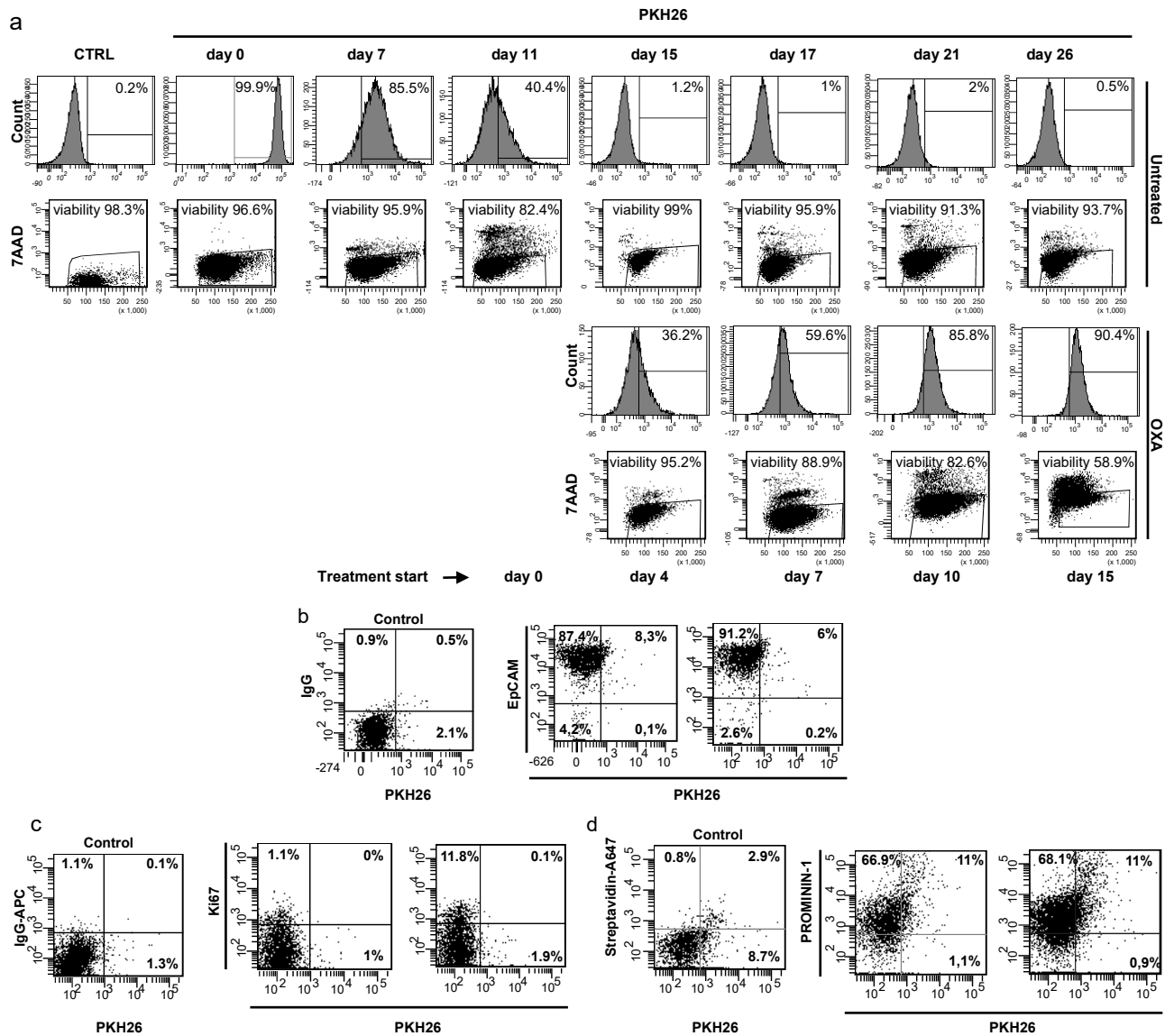

Supplementary Figure 1

Supplement: Supplementary file 4 — Additional file 4: Figure S1. Chemoresistance and marker expression of PKH26-positive cells. a Representative flow cytometry analysis of SW480 cells stained with PKH26 and treated with 2,5 μM oxaliplatin (OXA) starting from day 11, as described in Fig. 1a. Cell viability plots obtained with 7-AAD staining are shown below PKH26 plots, and the percentage of viable cells is indicated below each plot. b Representative flow cytometry analysis of EpCAM and PKH26 on xenograft-derived CCSCs 3 weeks upon subcutaneous injection of PKH26-stained cells in NSG mice. IgG, isotype control antibody. c Representative flow cytometry analysis of PKH26 and Ki67 in xenograft-derived EpCAM+ CCSCs at 3 weeks of tumor growth. d Representative flow cytometry analysis of PKH26 and PROMININ1 in xenograft-derived EpCAM+ CCSCs at 3 weeks of tumor growth. [file 13046_2019_1505_MOESM4_ESM.pdf]

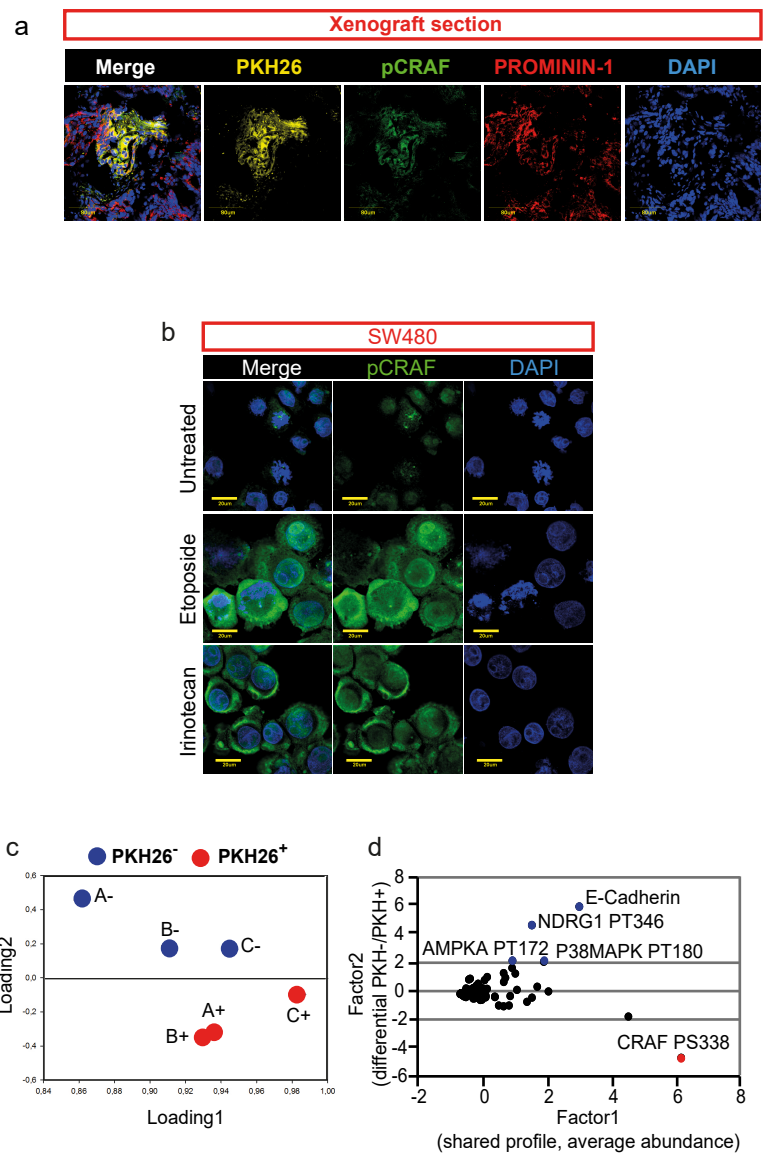

Supplementary Figure S2

Supplement: Supplementary file 8 — Additional file 8: Figure S2. Expression of pCRAF in vivo and in vitro and complementary RPPA data analysis. a Representative confocal microscopy images of PKH26-positive areas (yellow) in xenograft sections immunostained with anti-pCRAF S338 (green) and PROMININ1 (red). Scale bar, 80 μm. b Representative confocal microscopy images of SW480 cells treated for 48 h with 10 μM etoposide or 10 μM irinotecan and stained with anti-pCRAF S338 antibody. Scale bar, 20 μm. c Spatial representation of principal component (PC) analysis computed on a matrix having loading values of the two components, Factor 1 and Factor 2 that discriminates among PKH26+ and PKH26− samples. Results obtained on three PKH26+ versus PKH26− samples, n = 3 pools of 12 tumors each. d Spatial representation of scores of components representing relative RPPA antibodies values (Factor 1 and Factor 2). Results obtained on three PKH26+ versus PKH26− samples, n = 3 pools of 12 tumors each. [file 13046_2019_1505_MOESM8_ESM.pdf]

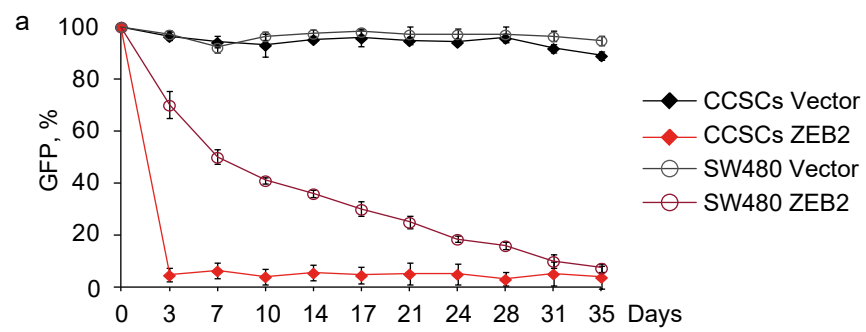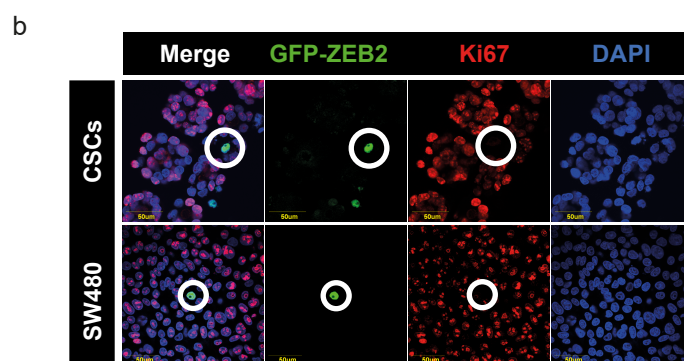

Supplementary Figure S3

Supplement: Supplementary file 9 — Additional file 9: Figure S3. Trends of ZEB2 overexpression in cultured cells. a Percentage of GFP positivity in CCSCs (diamonds) or SW480 (circles) cells transduced with empty pLenti-GFP (Vector) or with pLenti-GFP-ZEB2 (ZEB2) as assessed by flow cytometry for 5 weeks following lentiviral transduction and sorting (day 0). Graph shows the mean ± SD of three independent experiments. b Representative confocal image of CCSCs and SW480 cells transduced with GFP-ZEB2 and labeled with anti-Ki67 at day 28 after sorting. Circles indicate rare ZEB2+ cells, which are also Ki67−. Scale bar, 50 μm. [file 13046_2019_1505_MOESM9_ESM.pdf]

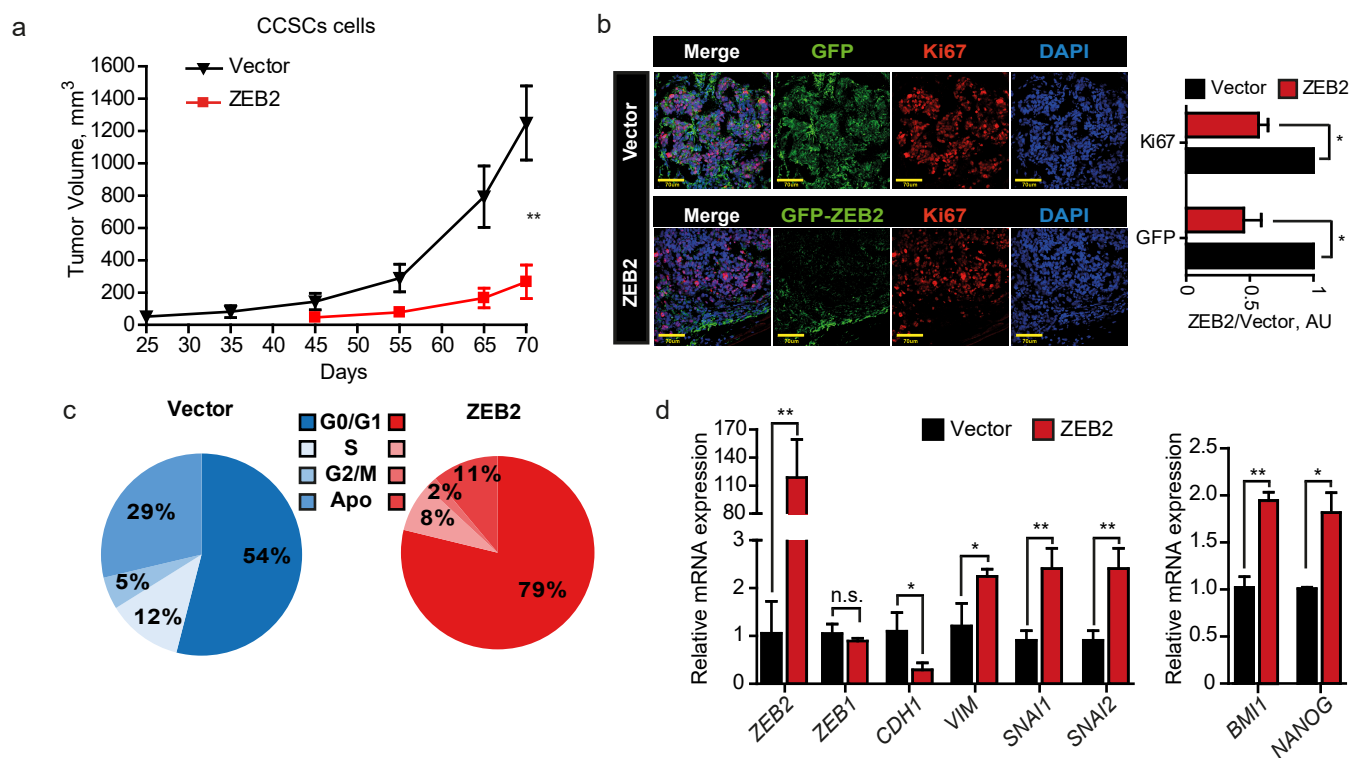

Supplementary Figure S4

Supplement: Supplementary file 10 — Additional file 10: Figure S4. In vivo effects of ZEB2 overexpression in CCSCs. a Volume of xenografts derived from CCSCs transduced with pLenti-GFP (Vector, black line/triangles) or with pLenti-GFP-ZEB2 (ZEB2, red line/squares). Graph shows the mean ± SEM, 6 tumors/group. **P < 0.01 from two-tailed t test. b Left panels: representative confocal images of xenograft sections derived from tumors obtained with primary cells transduced with pLenti-GFP (Vector) and GFP-ZEB2 (ZEB2). Sections were stained with anti-Ki67 (red). Scale bar, 70 μm. Quantification (right panel) was performed on 5 fields /group. *P < 0.05 from two-tailed t test. AU, arbitrary units. c Cell cycle analysis of GFP+ cells FACS-isolated from Vector and ZEB2-transduced tumors obtained with primary cells. d qRT-PCR analysis of the indicated transcripts in Vector- and ZEB2-transduced tumors obtained with primary cells, n = 3. *P < 0.05 and **P < 0.01 from two-tailed t test. n.s. = not significant by t test. Values are the mean ± SD. [file 13046_2019_1505_MOESM10_ESM.pdf]

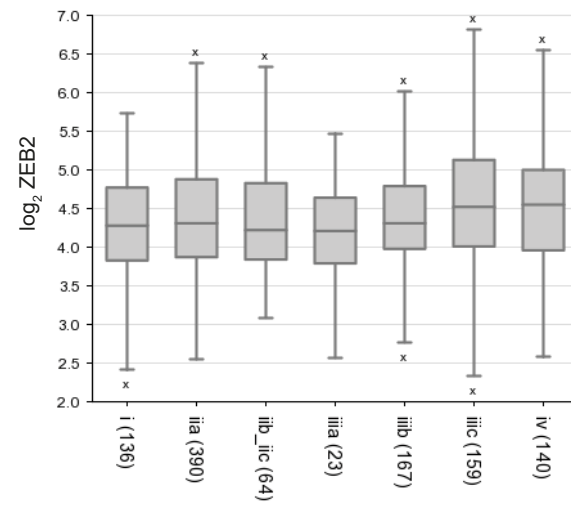

Supplementary Figure S5

Supplement: Supplementary file 11 — Additional file 11: Figure S5. ZEB2 expression in TNM stages, correlation with RFS and CMS in stage 2 CRC patients. ZEB2 transcript levels in the indicated number of CRC patients across all TNM stages. One-way ANOVA resulted in non-significant differences between stages. Outliers are depicted as crosses. n = 1079. [file 13046_2019_1505_MOESM11_ESM.pdf]
